# Leukemia Inhibitory Factor in Rat Fetal Lung Development: Expression and Functional Studies

**DOI:** 10.1371/journal.pone.0030517

**Published:** 2012-01-23

**Authors:** Cristina Nogueira-Silva, Paulina Piairo, Emanuel Carvalho-Dias, Francisca O. Peixoto, Rute S. Moura, Jorge Correia-Pinto

**Affiliations:** 1 Life and Health Sciences Research Institute (ICVS), School of Health Sciences, University of Minho, Braga, Portugal; 2 ICVS/3Bs - PT Government Associate Laboratory, Braga/Guimarães, Portugal; 3 Department of Obstetrics and Gynecology, Hospital de Braga, Braga, Portugal; 4 Department of Urology, Hospital de São João, Porto, Portugal; 5 Department of Pediatric Surgery, Hospital de Braga, Braga, Portugal; National Cancer Institute, United States of America

## Abstract

**Background:**

Leukemia inhibitory factor (LIF) and interleukin-6 (IL-6) are members of the family of the glycoprotein 130 (gp130)-type cytokines. These cytokines share gp130 as a common signal transducer, which explains why they show some functional redundancy. Recently, it was demonstrated that IL-6 promotes fetal lung branching. Additionally, LIF has been implicated in developmental processes of some branching organs. Thus, in this study LIF expression pattern and its effects on fetal rat lung morphogenesis were assessed.

**Methodology/Principal Findings:**

LIF and its subunit receptor LIFRα expression levels were evaluated by immunohistochemistry and western blot in fetal rat lungs of different gestational ages, ranging from 13.5 to 21.5 days post-conception. Throughout all gestational ages studied, LIF was constitutively expressed in pulmonary epithelium, whereas LIFRα was first mainly expressed in the mesenchyme, but after pseudoglandular stage it was also observed in epithelial cells. These results point to a LIF epithelium-mesenchyme cross-talk, which is known to be important for lung branching process. Regarding functional studies, fetal lung explants were cultured with increasing doses of LIF or LIF neutralizing antibodies during 4 days. MAPK, AKT, and STAT3 phosphorylation in the treated lung explants was analyzed. LIF supplementation significantly inhibited lung growth in spite of an increase in p44/42 phosphorylation. On the other hand, LIF inhibition significantly stimulated lung growth via p38 and Akt pathways.

**Conclusions/Significance:**

The present study describes that LIF and its subunit receptor LIFRα are constitutively expressed during fetal lung development and that they have an inhibitory physiological role on fetal lung branching.

## Introduction

Fetal lung development is a complex process, involving several effectors such as growth factors, extracellular matrix interactions, hormones and as, recently described inflammatory mediators [1]–[3]. In fact, it was already demonstrated that interleukin-6 (IL-6) promotes fetal lung maturation [4]–[6] and also that IL-6 is constitutively expressed in pulmonary primitive epithelium and enhances fetal lung branching [3].

IL-6 is one of the members of the family of the glycoprotein 130 (gp130)-type cytokines. This family comprises IL-6, leukemia inhibitory factor (LIF), IL-11, oncostatin M, ciliary neurotrophic factor (CNTF), cardiotropin-1 (CT-1) and cardiotrophin-like cytokine. These cytokines share the membrane glycoprotein gp130 as a common signal transducer which explains the fact that these show some functional redundancy even though they also exhibit specific biological activities [7], [8].

LIF is a pleiotropic cytokine, that exists in both soluble and matrix-bound forms, and that binds to a heterodimer LIF receptor alpha subunit (LIFRα)/gp130. Signal transduction involves the activation of Janus kinase (JAK) and the subsequent recruitment of signal transducers and activators of transcription (STAT) proteins, mainly STAT3. Alternatively, LIF can also initiate cell signaling via the mitogen-activated protein kinase (MAPK) cascade [7]–[10]. Moreover, LIF displays several biological activities ranging from the classic differentiation of myeloid leukemic cells into macrophage lineage to effects on proliferation of primordial germ cells, maintenance of embryonic stem cell pluripotentiality, endometrial decidualization, blastocyst implantation, neural development, bone cell metabolism, adipocyte lipid and energy homeostasis, muscle satellite cell proliferation, heart hypertrophy, inhibition of retinal vascularization and inflammation [9]–[13]. Furthermore, several studies have emphasized the importance of LIF signaling in several processes of branching organs. For instance, this cytokine inhibits fetal nephrons formation [14]–[16], induces mammary gland involution [17], decreases thyroid tumors growth [18], [19] and increases pancreatic regeneration [20]. It was already described that LIF, together with insulin-like growth factor I (IGF-I), regulates lung maturation. In fact, absence of LIF in addition to IGF-I null mutant mice aggravates pulmonary immaturity. Indeed, LIF/IGF-I double deficient embryos present lung hypoplasia and defective differentiation of the alveolar epithelium and vasculogenesis [21].

Regardless of several evidences in literature that point towards a possible involvement of LIF during fetal lung development, LIF expression pattern as well as its effects during lung morphogenesis are largely unknown. Despite LIF/IGF-I double deficient mice lung phenotype, LIF knockout mice have no significant abnormal lung features [21], [22]. However, in the current study, it was demonstrated that LIF and LIFRα were constitutively expressed during fetal lung development and that in vitro LIF supplementation significantly inhibited lung growth, likely through p44/42 pathway.

## Results

### LIF and LIFRα expression pattern during fetal lung development

**Figure 1:**
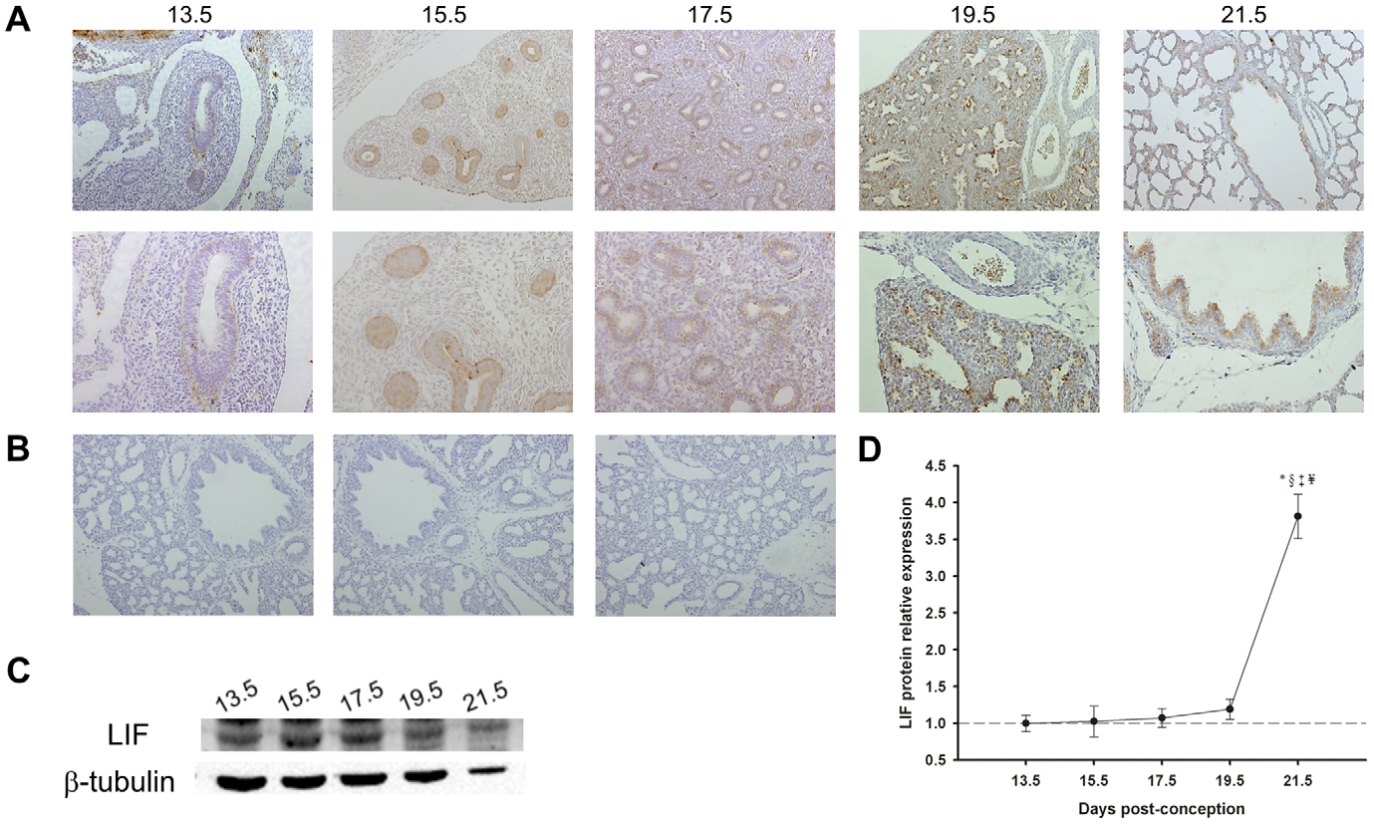
LIF expression pattern during fetal lung development (from 13.5 until 21.5 dpc). (A) IHC studies revealed that LIF expression was localized to airway epithelium. Original magnification: Upper panel – ×100; Bottom panel – ×200. (B) IHC negative controls: Left – omission of the primary antibody; Center – non-immune goat IgG isotype control; Right – simultaneous omission of the primary and secondary antibodies. In all negative controls immunoreactive LIF staining was not observed. (C) Western blot analysis of LIF throughout gestation (45 kDa). Control loading was performed using β-tubulin (55 kDa). (D) Relative LIF protein levels expressed in arbitrary units normalized for β-tubulin. p<0.05: ^*^ *vs.* 13.5 dpc, ^§^ *vs.* 15.5 dpc, ^‡^ *vs.* 17.5 dpc, ^¥^ *vs.* 19.5 dpc.

**Figure 2:**
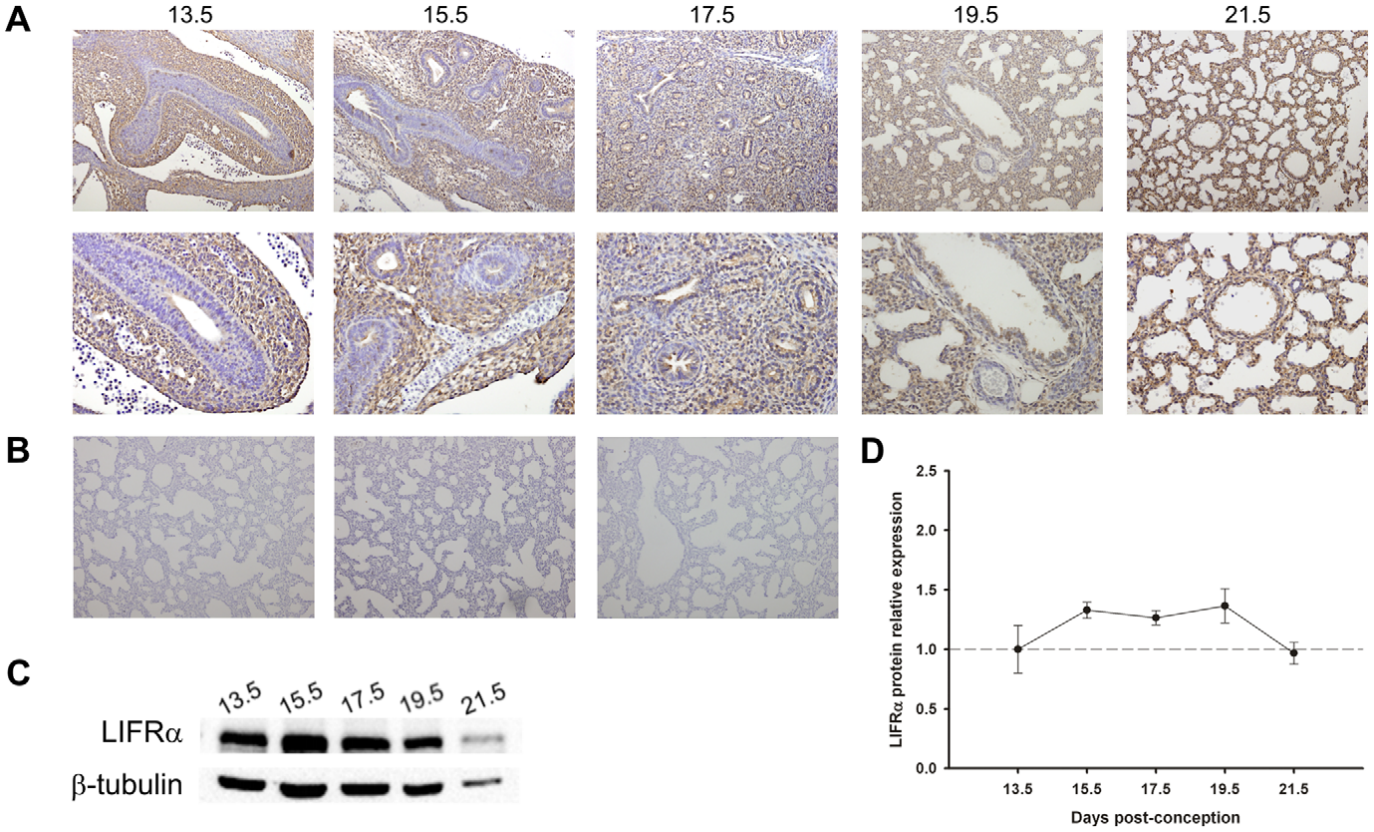
LIFRα expression pattern during fetal lung development (from 13.5 until 21.5 dpc). (A) IHC studies revealed that LIFRα was first mainly expressed in the mesenchyme, but after pseudoglandular stage it was also observed in epithelial cells throughout gestation. Original magnification: Upper panel – ×100; Bottom panel – ×200. (B) IHC negative controls: Left – omission of the primary antibody; Center – non-immune goat IgG isotype control; Right – simultaneous omission of the primary and secondary antibodies. In all negative controls immunoreactive LIFRα staining was not observed. (C) Western blot analysis of LIFRα throughout the gestation (190 kDa). Control loading was performed using β-tubulin (55 kDa). (D) Relative LIFRα protein levels expressed in arbitrary units normalized for β-tubulin. No significant difference was observed between gestational ages.

The immunohistochemistry (IHC) and western blot studies revealed that LIF and LIFRα protein were expressed throughout all studied gestational ages in fetal lung (Figure 1 and 2). LIF was mainly expressed in bronchiolar and also in alveolar epithelium from 13.5 days post-conception (dpc) until term. At 19.5 and 21.5 dpc, mesenchymal tissue also displayed scattered LIF positive cells (Figure 1A and 1B). These LIF expression results prompted us to perform a western blot analysis in order to quantify the relative expression of LIF levels during normal lung development. LIF expression levels were minimal at 13.5 dpc, increased slightly throughout gestation and reached its maximum at the end of gestation (21.5 dpc) (Figure 1C and 1D).

Regarding LIFRα, early in gestation, protein expression was restricted to the mesenchyme (Figure 2A and 2B). Throughout fetal lung development, namely from 17.5 dpc onwards, LIFRα mesenchymal expression decreased and epithelial expression was observed until term (Figure 2A and 2B). LIFRα expression levels remained constant during fetal lung development (Figure 2C and 2D).

### Role of LIF in fetal lung development

**Figure 3:**
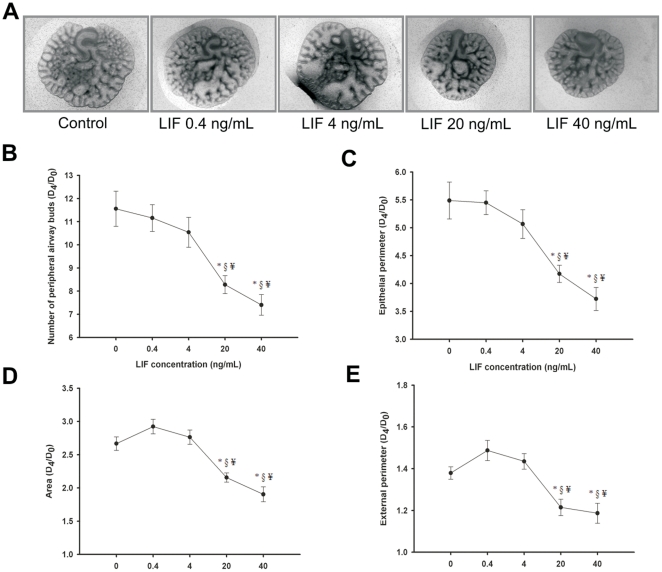
LIF supplementation studies in a fetal lung explant culture system. (A) Representative examples of fetal lung explants treated daily with increasing concentrations of recombinant LIF, after 4 days in culture. Original magnification ×25. (B) Number of total airway buds; (C) Epithelial perimeter; (D) Area; (E) External perimeter of lung explants treated with LIF. LIF significantly inhibited lung growth. Results are expressed as ratio of day 4 (D_4_) and day 0 (D_0_) of culture (D_4_/D_0_ ratio). p<0.05: ^*^ *vs.* LIF at 0 ng/mL (control), ^§^ *vs.* LIF at 0.4 ng/mL, ^¥^ vs. LIF at 4 ng/mL.

In order to evaluate the role of LIF on lung morphogenesis, fetal lung explants were treated daily with increasing concentrations of recombinant LIF. In Figure 3A, representative examples of fetal lung explants treated with increasing LIF concentrations, after 4 days in culture, are illustrated. LIF appears to have a dose-effect inhibitory action on lung explants growth. In fact, a decrease in the total number of peripheral airway buds (Figure 3B), epithelial perimeter (Figure 3C), area (Figure 3D) and external perimeter (Figure 3E) of lung explants was observed in all concentrations tested, this effect is most significant in the highest LIF concentrations studied, 20 and 40 ng/mL.

**Figure 4:**
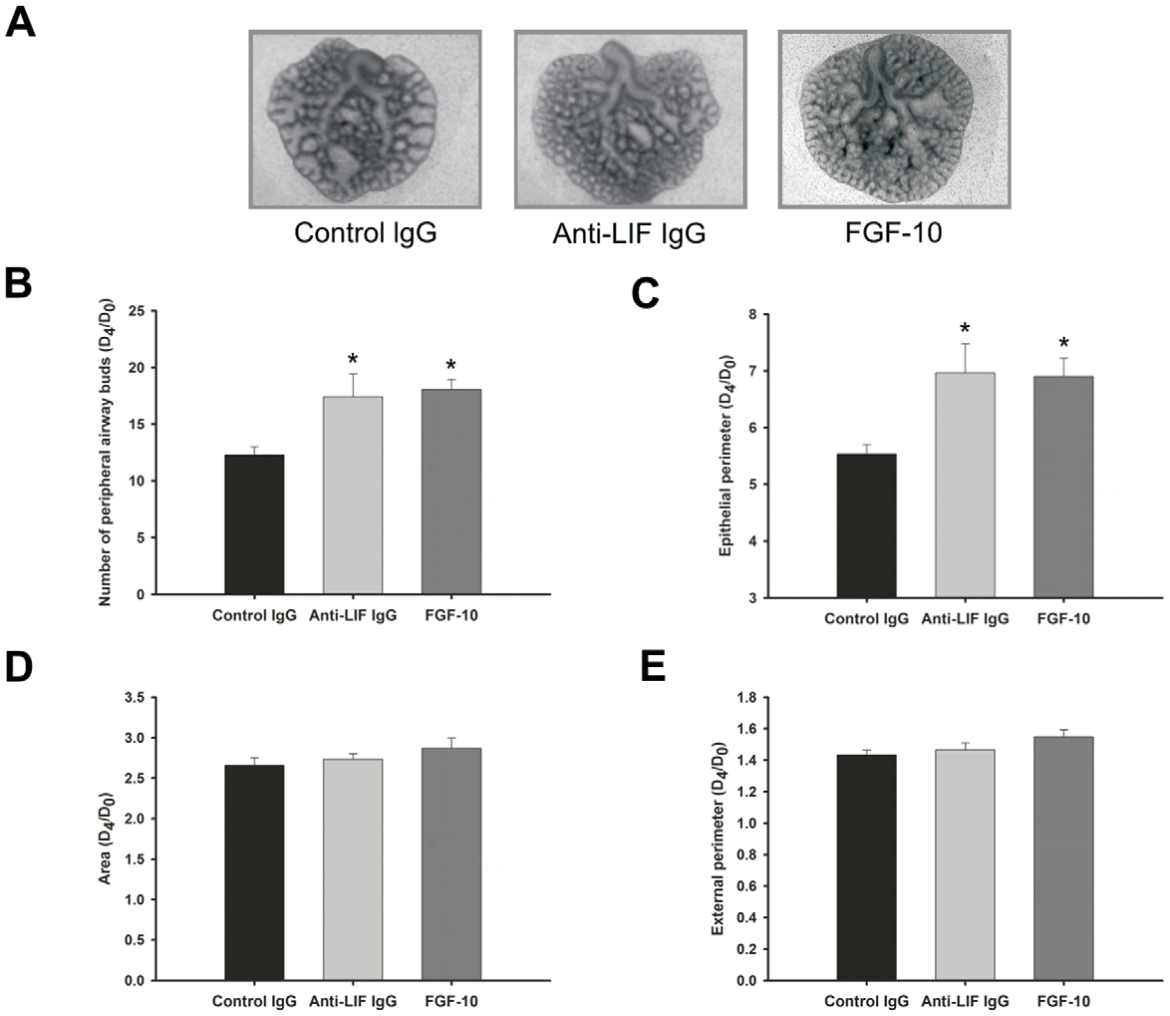
LIF neutralizing studies in a fetal lung explant culture system. (A) Representative examples of lung explants treated daily with normal IgG (control; 1 µg/mL), anti-LIF IgG (1 µg/mL) or FGF-10 (500 ng/mL), after 4 days in culture. Original magnification ×25. (B) Number of total airway buds; (C) Epithelial perimeter; (D) Area; (E) External perimeter of treated lung explants. Inhibition of LIF action significantly stimulated lung branching in a similar way than FGF-10. Results are expressed as D_4_/D_0_ ratio. p<0.05: ^*^ *vs.* control IgG.

In Figure 4 it is shown the morphometric analysis of lung explants treated with control IgG, anti-LIF IgG antibodies or FGF-10. LIF inhibition significantly stimulated lung branching (Figure 4B) and epithelial perimeter (Figure 4C) in a similar way as FGF-10. No differences were observed on lung explant area (Figure 4D) or external perimeter (Figure 4E).

**Figure 5:**
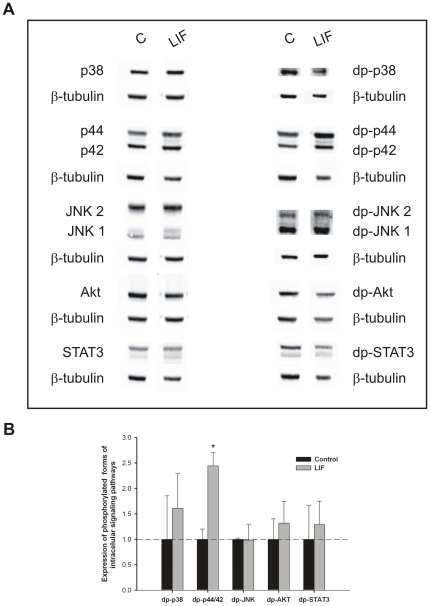
Analysis of intracellular signaling pathways that mediates LIF actions on lung growth. (A) Western blot analysis of p38, p44/42, JNK1/2, Akt and STAT3, and to diphosphorylated forms of p38 (dp-p38), p44/42 (dp-p44/42), SAPK/JNK (dp-JNK1/2), Akt (dp-Akt) and STAT3 (dp-STAT3) in control lung explants (C) and treated with LIF at 40 ng/mL (LIF). Control loading was performed using β-tubulin (55 kDa). p38 corresponds to 38 kDa. p44/42 correspond to 44 and 42 kDa, respectively. JNK1 and 2 correspond to 46 and 54 kDa, respectively. Akt corresponds to 60 kDa. STAT3 corresponds to two bands, 79 and 86 kDa. (B) Semi-quantitative analysis of expression of phosphorylated forms of these intracellular signaling pathways. Results are presented as arbitrary units normalized for β-tubulin. p<0.05: ^*^ *vs.* control.

**Figure 6:**
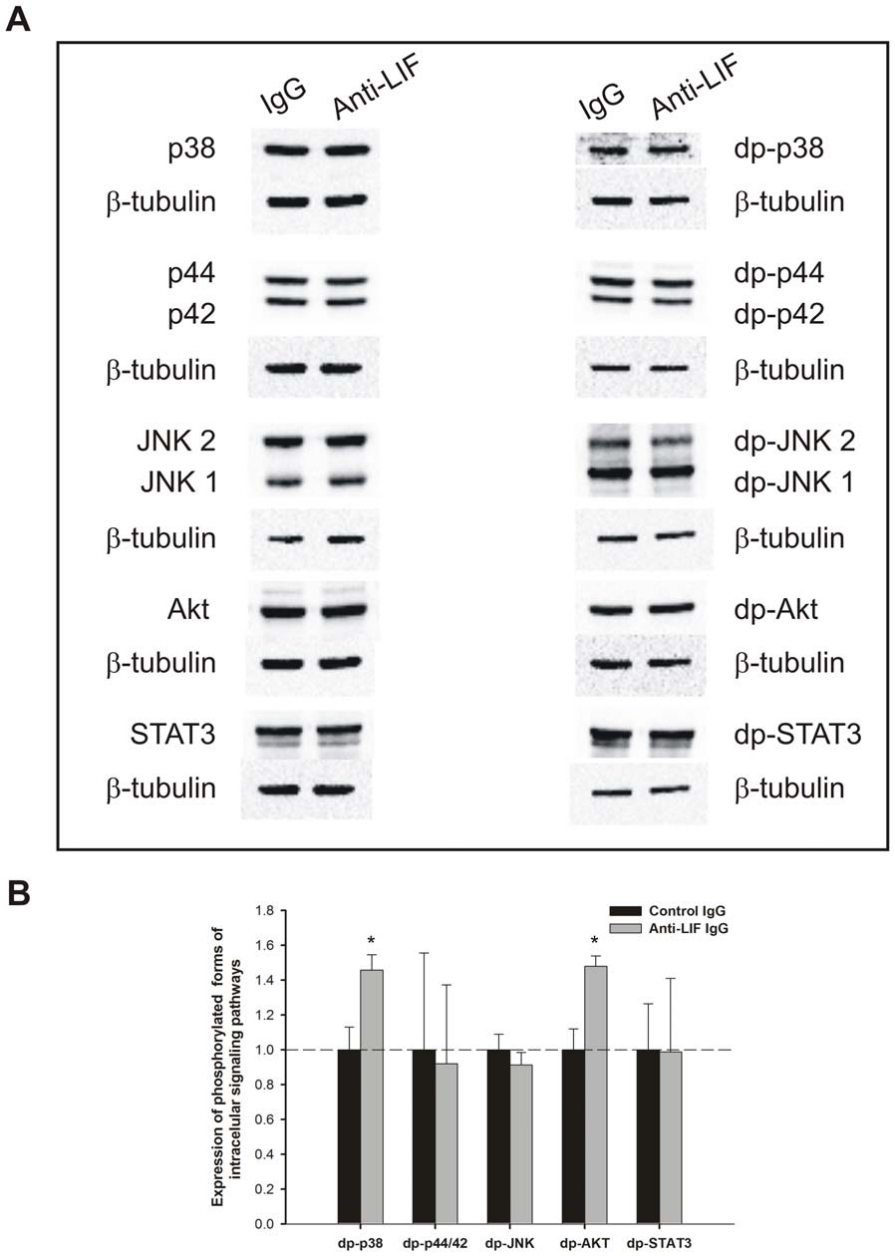
Analysis of intracellular signaling pathways that mediates anti-LIF actions on lung branching. (A) Western blot analysis of p38, p44/42, JNK1/2, Akt and STAT3, and to diphosphorylated forms of p38 (dp-p38), p44/42 (dp-p44/42), SAPK/JNK (dp-JNK1/2), Akt (dp-Akt) and STAT3 (dp-STAT3) in control IgG lung explants (IgG) and treated with anti-LIF IgG (Anti-LIF). Control loading was performed using β-tubulin (55 kDa). (B) Semi-quantitative analysis of expression of phosphorylated forms of these intracellular signaling pathways. Results are presented as arbitrary units normalized for β-tubulin. p<0.05: ^*^ *vs.* IgG.

Pooled samples of lung explants (n = 15) treated with LIF at 40 ng/mL (selected due to its maximal effect on inhibition of explants growth) were evaluated for modulation of MAPK, Akt and STAT3 pathways (Figure 5). Lung growth inhibition induced by LIF significantly stimulated p44/42 phosphorylation (Figure 5). On the other hand, the increase on lung branching induced by blocking LIF action significantly stimulated p38 and Akt phosphorylation (Figure 6).

## Discussion

This study demonstrated that LIF was constitutively expressed in pulmonary epithelium, whereas its subunit receptor, LIFRα, was expressed in the mesenchyme until the pseudoglandular stage and in epithelial cells, at later stages. Moreover, LIF supplementation significantly inhibited lung growth most likely through p44/42 pathway. On the other hand, LIF inhibition significantly increased lung branching and stimulated p38 and Akt phosphorylation.

LIF is a multifunctional glycoprotein cytokine that regulates many cellular responses such as proliferation, differentiation and survival in different cellular types [8]–[13]. Regarding the lung, several studies have investigated LIF's role in the pathophysiology of different lung diseases, such as acute respiratory distress syndrome [23], asthma [24], chronic airway inflammation [25] and inflammation induced by tobacco [23]. In fact, LIF is well known to induce the synthesis and activation of phospholipase A_2_ in bronchial epithelial cells [12] and it has protective effects in the setting of hyperoxic lung injury [26]. Concerning LIF's putative role during prenatal lung period, all previous studies had focused mainly in maturation processes.

In the current study, it was demonstrated that LIF was expressed in primitive lung epithelium, since early stages of lung development (13.5 dpc), a moment in which predominantly occurs growth phenomena. LIF expression in bronchiolar and also in alveolar epithelium is in agreement with previous findings documented in adult human lung [25], [27], [28]. In adults, epithelial expression was also observed in other branching organs such as mammary gland [29] and pancreas [20]. During murine fetal development, LIF expression was already described in the ureteric bud [15], skin, skeletal muscle, heart, brain, liver and gut [30], [31] supporting a role for this cytokine in the normal development of several organ systems. Moreover, LIF expression was observed in all studied gestational ages and its expression levels increased by the end of the gestation, which is in accordance with the literature [31].

Regarding LIFRα, it was expressed in constant levels throughout gestation: first mainly in lung mesenchyme, and after pseudoglandular stage it was predominantly observed in the epithelium until term. LIFRα is known to be expressed in numerous human lung structural cell types, namely fibroblasts, bronchial smooth muscle cells and airway epithelial cells [25], [27], [28]. Interestingly, during fetal kidney morphogenesis, LIFRα was first detected in metanephric mesenchyme and later in newly formed tubules [15]. It is necessary to stress that the occurrence of epithelial LIF and mesenchymal LIFRα expression, since early stages of lung development and until the end of pseudoglandular stage, points to a LIF epithelium-mesenchyme interaction during this stage of lung development, which is mainly characterized by successive branching phenomena. It is well described that lung development occurs through specific cross-talk interactions between epithelium and mesenchyme [1]. Therefore, LIF and LIFRα interaction might possibly be a new player on lung branching mechanisms. Nonetheless, the highest LIF expression was observed at the end of the gestation which also suggests a physiological role for LIF in lung maturation, as already demonstrated in literature [21], [22].

In order to evaluate LIF's role in lung branching morphogenesis, fetal lung explants were cultured with increasing doses of LIF or LIF neutralizing antibodies. LIF supplementation inhibited lung growth, whereas LIF blockage stimulated lung branching. Interestingly, the blockage of LIF activity increased the number of peripheral airways buds and epithelial perimeter in a similar way as FGF-10, a classical and very important lung growth factor that is known to increase lung branching [32], [33]. LIF inhibitory effect had already been described in fetal kidney development [16] and in the proliferation of human normal and malign breast epithelial cells [34] which, like the lung, develop by branching events. Moreover, LIF inhibitory effect was also described on other cell types such as embryonic stem cells [35], bone [36], endothelial [11] and neuronal cells lines [37]. However, LIF stimulates duct cell proliferation in adult pancreas [18]. Interestingly, this LIF inhibitory effect on fetal lung branching opposes the stimulatory effect described for IL-6 [3], also a member of family of the gp130-type cytokines.

Although this *in vitro* study clearly suggests a physiological role for LIF on lung branching, LIF knockout mice do not seem to have abnormal lung features when compared to normal littermates [21], [22]. Indeed, LIF knockout mice are born normal and they only exhibit defective blastocyst implantation, postnatal growth retardation and hematopoiesis defects [22], [38], [39]. The absence of significant lung development abnormalities in LIF knockout mice does not mean a minor role for LIF during lung morphogenesis *in vivo*. In fact, as demonstrated for several phenomena, mice lacking individual members of gp130-type cytokines displayed milder phenotype than expected, which is most likely due to the similarity and redundancy of downstream events induced by gp130-dependent cytokines [38]. On the other hand, LIFRα deletion causes perinatal death with multiple defects [22], [38], [40]–[42]. However, it is necessary to stress that this receptor subunit is a part of the receptor complexes for LIF, CNTF and CT-1 [8]. Moreover, it is necessary to stress that it is recognized that the knockout approach also bears intrinsic problems, since the ensuing phenotype may consist of compensatory events during development and later life [43]. In opposition to LIF-deficient lungs, the additional absence of LIF in the IGF-I-null background of mutant mice aggravates the prenatal pulmonary immature phenotype and these mice die due to respiratory failure [21], [22]. These results point to a possible LIF role on lung maturation, but only involving also IGF-I. Furthermore, lung development is a complex process involving two different cellular phenomena, maturation and growth [1], and in the context of present work LIF's involvement was studied with regard to the growth phenomena.

In different cells and tissues, LIF signaling is mediated mainly by JAK/STAT3, p44/42 kinase and PI3K/Akt pathways [7]–[10], [17]. Interestingly, many of the effectors that modulate fetal lung branching morphogenesis seem to activate signaling pathways that converge into the MAPK and PI3K/Akt cascades [44]. Thus, MAPK, PI3K/Akt and STAT3 pathway modulation by LIF and anti-LIF in fetal lung development was investigated. The inhibition of lung growth induced by LIF significantly stimulated p44/42 phosphorylation and did not significantly change p38, JNK, Akt or STAT3 pathways. In contrast to these results, in lung development, the p44/42 pathway has been involved in stimulation of branching morphogenesis. Indeed, Kling *et al* demonstrated that p44/42 inhibition reduces branching morphogenesis and causes mesenchymal cell apoptosis in fetal rat lungs [44]. According to the literature, it is not the first association between LIF, p44/42 pathway and growth inhibition/arrest. In fact, in human medullary thyroid cancer cells, it was demonstrated that p44/42 pathway induces autocrine-paracrine growth inhibition via LIF [19]. Moreover, the absence of LIF in the IGF-1 null mice, which induces changes on pulmonary maturation and vasculogenesis, also induces activation of p44/42 [21]. Furthermore, the LIF action mediated by p44/42 was already demonstrated in earlier mammary gland development [17] and myoblasts [45]. Thus, considering that lung explants were incubated during 4 days, p44/42 stimulation might not be the direct pathway that mediates LIF action, but the result of crosstalks of other non-canonical intracellular pathway. Regarding anti-LIF treatment, the increase on lung branching observed was mediated by stimulation of p38 and Akt phosphorylation. PI3K/Akt pathway is a classical stimulator of lung branching [44], [46]. Moreover, p38 was already demonstrated as mediator of lung branching [3], [47]. Interestingly, it was demonstrated that IL-6, another a gp130-type cytokines, also enhances lung branching via p38 phosphorylation [3].

In this study, it was demonstrated that LIF inhibits lung branching, in opposition to IL-6 (another cytokine of the gp130 family). Although cytokines of the gp130 family share a common signal transducer, these findings suggest specific biological activities for each cytokine on lung development. In fact, specific characteristics are emerging for each member of this family, brought about mainly through restricted temporal and spatial release, differential expression of cell surface receptors, and different signaling patterns between gp130 homodimers and heterodimers [25]. Interestingly, LIF receptor is a gp130 heterodimer, whereas IL-6 receptor is a gp130 homodimer. Thus, this raises the hypothesis that the effect, on lung development, of cytokine signaling through gp130 heterodimers might be different and even opposite to gp130 homodimers. Although this hypothesis needs to be verified, this specificity can possibly represent a regulatory mechanism of lung morphogenesis, intrinsic to this family of cytokines, in order to achieve the correct lung growth.

In conclusion, LIF and LIFRα expression during fetal lung development as well as the *in vitro* studies presented in this work suggest an inhibitory physiological role for endogenous LIF, mediated by epithelial-mesenchymal interactions, on pulmonary branching mechanisms.

## Materials and Methods

This study was carried out in strict accordance with the recommendations in the ‘Guide for the Care and Use of Laboratory Animals’, published by the US National Institutes of Health (NIH Publication No.85-23, revised 1996). Animal experiments were also performed according to the Portuguese law for animal welfare and the protocol was approved by the Committee on the Ethics of Animal Experiments of the Life and Health Sciences Research Institute of the University of Minho (DGV 022162 - 520/000/000/2006). Moreover, all efforts were made to minimize animal suffering.

### Animal model and experimental design

Sprague-Dawley female rats (225 g; Charles-River, Spain) were maintained in appropriate cages under temperature-controlled room (22–23°C) on a 12 hours light: 12 hours dark cycle, and fed with commercial solid food. The rats were mated and checked daily for vaginal plug. The day of plugging was defined as gestational day 0.5 for time dating purposes. Fetuses were removed by caesarean section at 13.5, 15.5, 17.5, 19.5 and 21.5 dpc (days post-conception) and sacrificed by decapitation. Lungs were dissected and processed for IHC and western blot analysis. To perform fetal lung explant cultures fetuses were harvested at 13.5 dpc and their lungs dissected.

### IHC

LIF and LIFRα immunostainings were performed on formalin-fixed and paraffin-embedded lungs of different gestational ages (13.5–21.5dpc). Sections (5 µm) were placed on SuperFrost®Plus slides (Menzel-Glaser, Germany). LIF antibody (sc-1336; Santa Cruz Biotechnology Inc., USA) was used in a 1∶50 dilution and LIFRα antibody (sc-659; Santa Cruz Biotechnology Inc.) in a 1∶50 dilution. After dewaxing in xylene and rehydration in ethanol, the samples were incubated in 3% hydrogen peroxide in methanol to quench endogenous peroxidase. Antigen retrieval was achieved by boiling in 10 mM citrate buffer followed by cool down at room temperature. Samples were blocked with 5% BSA (Roche, Germany). Incubation of the primary antibody occurred at 4°C overnight. Negative control reactions included omission of the primary antibody, the simultaneous omission of the primary and secondary antibodies, and a non-immune goat IgG isotype control diluted to a matching concentration as the primary antibody, instead of the primary antibody. Incubation with the goat ImmunoCruz™ Staining System (sc-2053; Santa Cruz Biotechnology Inc.) or with the UltraVision detection system anti-polyvalent horseradish peroxidase (Lab Vision Corporation, USA) was carried according to manufacturer's instructions (for LIF and LIFR immunostainings, respectively). To visualize the peroxidase activities in sections, diaminobenzidine tetrahydrochloride (Dako, Denmark) was used. Sections were counterstained with hematoxyline. The slides were observed and photographed with Olympus BX61 microscope (Olympus, Japan). The pictures presented are representative of 6 animals (n = 6) and 12 samples for each gestational age, and three independent experiments were performed.

### Western blot analysis

Different pooled lung samples for each gestational age (13.5–21.5 dpc) and cultured lung explants were processed for western blot analysis. Proteins were obtained according to Kling *et al* [44]. Ten or twenty five µg of protein were loaded onto 10% or 7.5% acrylamide minigels (respectively), electrophoresed at 100 V at room temperature and then transferred to nitrocellulose membranes (Hybond™ -C Extra, GE Healthcare Life Sciences, UK). Blots were probed with LIF and LIFRα polyclonal antibodies (Santa Cruz Biotechnology Inc.) according to manufacturer's instructions (1∶250 and 1∶500, respectively). For loading control, blots were probed with β-tubulin MAb (1∶200000, Abcam, UK). Afterwards blots were incubated with a secondary horseradish peroxidase conjugate (Santa Cruz Biotechnology Inc.), developed with Super Signal®West Femto Substrate (Pierce Biotechnology, USA) and the chemiluminescent signal was captured using the Chemidoc XRS (Bio-Rad, USA). Quantitative analysis was performed with Quantity One 4.6.5 1-D Analysis Software (Bio-Rad). Three independent experiments were performed (n = 3).

### Fetal lung explant cultures

Harvesting and dissection of 13.5 dpc lungs was made in DPBS (Lonza, Switzerland) under a dissection microscope (Leica MZFLIII, Switzerland). The lungs were transferred to Nucleopore membranes with an 8 µm pore size (Whatman, USA) and incubated in a 24-well culture plates (Nunc, Denmark). The membranes were pre-soaked in DMEM (Invitrogen, UK) for 1 hour before the explants were placed on them. Floating cultures of the explants were incubated in 200 µl of 50% DMEM, 50% nutrient mixture F-12 (Gibco, USA) supplemented with 100 µg/ml streptomycin, 100 units/ml penicillin (Gibco), 0.25 mg/mL ascorbic acid (Sigma-Aldrich, USA) and 10% FCS (Gibco). The fetal lung explants were incubated in a 5% CO_2_ incubator at 37°C for 96 hours, and the medium was replaced every 48 hours. The branching morphogenesis was monitored daily by photographing the explants. At day 0 (D_0_: 0 hours) and day 4 (D_4_: 96 hours) of culture, the total number of peripheral airway buds (branching) in all lung explants was determined, whereas the explants area, epithelial perimeter and external perimeter were measured using AxionVision Rel. 4.3 (Carl Zeiss, Germany). These results were expressed as D_4_/D_0_ ratio.

### LIF supplementation studies

Twelve lung explants were used as control (0 ng/mL). Additionally, recombinant LIF (Sigma-Aldrich) was daily added to lung explants in order to achieve a final concentration of 0.4, 4, 20 and 40 ng/mL (0.4 n = 15; 4 n = 12; 20 n = 12; 40 n = 15).

After 4 days in culture, control and LIF treated lung explants (at 40 ng/mL) were processed for western blot analysis of non-phosphorylated and phosphorylated forms of p38, p44/42, JNK, Akt and STAT3 (Cell Signaling Technology Inc., USA) according to the method described above. Four independent experiments were performed (n = 4).

### LIF neutralizing studies

Lung explants were treated daily with IgG anti-LIF neutralizing antibody (n = 12) at concentration of 1.0 µg/mL (R&D Systems, USA). Non-specific effects were evaluated by daily addition of identical concentration of normal IgG control antibody (n = 9; R&D Systems). Moreover, lung explants were also treated with FGF-10 that was added daily to the culture medium at a concentration of 500 ng/mL (n = 9; R&D Systems).

After 4 days in culture, normal IgG and anti-LIF IgG treated lung explants were processed for western blot analysis of the intracellular signaling pathways, as described above (n = 4).

### Statistical Analysis

All quantitative data are presented as mean ± SEM. Statistical analysis was performed using the statistical software SigmaStat (version 3.5; Systat Software Inc., USA). For LIF and LIFRα expression levels evaluation and supplementation studies one-way ANOVA was used. For LIF neutralizing studies and intracellular signaling pathways analysis *t*-test analysis was used. The Student-Newman-Keuls test was used for post-test analysis. Statistical significance was set at p<0.05.
